# H19 activates Wnt signaling and promotes osteoblast differentiation by functioning as a competing endogenous RNA

**DOI:** 10.1038/srep20121

**Published:** 2016-02-08

**Authors:** Wei-Cheng Liang, Wei-Ming Fu, Yu-Bing Wang, Yu-Xin Sun, Liang-Liang Xu, Cheuk-Wa Wong, Kai-Ming Chan, Gang Li, Mary Miu-Yee Waye, Jin-Fang Zhang

**Affiliations:** 1 School of Pharmaceutical Sciences, Southern Medical University, Guangzhou 510515, P.R.China; 2 School of Biomedical Sciences, The Chinese University of Hong Kong, Shatin, New Territories, Hong Kong, P.R. China; 3 Guangzhou Institute of Advanced Technology, Chinese Academy of Sciences, Guangzhou, 510000, P.R. China; 4 Department of Orthopaedics & Traumatology, The Chinese University of Hong Kong, Prince of Wales Hospital, Shatin, Hong Kong, P.R. China; 5 Shenzhen Research Institute, The Chinese University of Hong Kong, Shenzhen, P.R. China; 6 Stem Cells and Regenerative Medicine Laboratory, Lui Che Woo Institute of Innovative Medicine, Li Ka Shing Institute of Health Sciences, The Chinese University of Hong Kong, Prince of Wales Hospital, Shatin, Hong Kong, P.R. China; 7 School of medicine, South China Unversity of Technlogy, Guangzhou, 510000, P.R. China

## Abstract

Bone homeostasis is tightly orchestrated and maintained by the balance between osteoblasts and osteoclasts. Recent studies have greatly expanded our understanding of the molecular mechanisms of cellular differentiation. However, the functional roles of non-coding RNAs particularly lncRNAs in remodeling bone architecture remain elusive. In our study, lncRNA H19 was found to be upregulated during osteogenesis in hMSCs. Stable expression of H19 significantly accelerated *in vivo* and *in vitro* osteoblast differentiation. Meanwhile, by using bioinformatic investigations and RIP assays combined with luciferase reporter assays, we demonstrated that H19 functioned as an miRNA sponge for miR-141 and miR-22, both of which were negative regulators of osteogenesis and Wnt/β-catenin pathway. Further investigations revealed that H19 antagonized the functions of these two miRNAs and led to de-repression of their shared target gene β-catenin, which eventually activated Wnt/β-catenin pathway and hence potentiated osteogenesis. In addition, we also identified a novel regulatory feedback loop between H19 and its encoded miR-675-5p. And miR-675-5p was found to directly target H19 and counteracted osteoblast differentiation. To sum up, these observations indicate that the lncRNA H19 modulates Wnt/β-catenin pathway by acting as a competing endogenous RNA, which may shed light on the functional role of lncRNAs in coordinating osteogenesis.

Bone is continuously being remodeled in a dynamic balance where osteoblasts produce new bone tissue while osteoclasts destroy and resorb bone tissues^1^. As a major constituent of bone, osteoblasts are essential for maintaining skeletal architecture and modulating bone microenvironment homeostasis. It is well documented that osteoblasts produce a variety of extracellular matrix proteins, including OCN, ALP, OPN and Type I collagen^2^. These extracellular matrix proteins are fundamental for the maintenance of bone homeostasis and disruption of bone matrix deposition would eventually lead to a number of bone diseases such as osteoporosis and osteogenesis imperfect^3^. Hence, the understanding of the molecular regulatory networks of osteoblast differentiation is crucial for developing therapeutic tools for the treatment of bone diseases.

As a vital origin of osteoblast, hMSCs are stromal cells with multipotency, which can fully differentiate towards various cell types such as osteoblasts, chondrocytes and adipocytes^4^,^5^. During the osteoblast differentiation, numerous cytokines and growth factors have been identified to play an important role in regulating osteoblast replication and cellular differentiation. For instance, several BMPs, particularly BMP2, can initiate osteogenesis by providing extracellular signals and subsequently trigger mineral deposition^6^,^7^. In addition to BMPs, TGF-β1 also plays an important role in regulating bone mass and TGF-β1 deficient mice was found to exhibit reduced bone growth and impaired mineralization capacity^8^.

In the past decade, lncRNAs emerge as novel regulators of numerous biological processes, such as transcriptional regulation, cancer progression, and cellular differentiation^9^,^10^. In the past decade, a few high throughput technologies have been applied to the investigation of lncRNA expression profiles during osteoblast differentiation, which have successfully characterized a small number of osteogenesis-related lncRNAs^11^. For example, lncRNA-ANCR were found to accelerate osteogenesis by physically interacting with EZH2 and directly modulating Runx2 expression^12^. Nevertheless, in spite of promising achievements, currently the functional role of lncRNA in regulating osteogenesis is still poorly understood.

Recent experimental investigations have highlighted that, under some circumstances, RNA transcripts may affect each other’s RNA levels by competing for a limited number of miRNAs^13^. To synthesize these recent findings, the competing endogenous RNA hypothesis (ceRNA hypothesis) was put forward and this hypothesis elucidated a previously unknown role of lncRNAs in regulating other RNA transcripts’ function. According to the ceRNA hypothesis, all types of RNA transcripts (no matter lncRNAs or protein-coding RNAs) communicate with each other by competing for shared miRNAs through the miRNA binding sites. Hence, the expression alteration of lncRNAs may lead to the disruption of protein-coding gene networks and thus coordinates a number of important cellular events including bone formation and remodeling.

The lncRNA H19, one of the most well-known imprinted genes, is located on human chromosome 11 and it is transcribed only from the maternally inherited allele^14^. In the past twenty years, a broad spectrum of studies have been conducted to evaluate the function of H19 in genomic imprinting. Recent research studies have highlighted H19 as an active modulator in embryonic placental growth and skeletal muscle differentitation^15^,^16^. However, unfortunately, the role of H19 in osteoblast differentiation is largely unknown and its function remains to be characterized. In the present study, we identified H19 as a novel activator of Wnt/β-catenin pathway by serving as an miRNA sponge, which eventually promoted the cellular differentiation from hMSCs towards mature osteoblast. Meanwhile, we also characterized a novel feedback loop between H19 and its encoded miR-675-5p and elucidated this regulatory axis in osteoblast differentiation.

## Results

### LncRNA H19 positively regulates osteoblast differentiation

**Figure 1:**
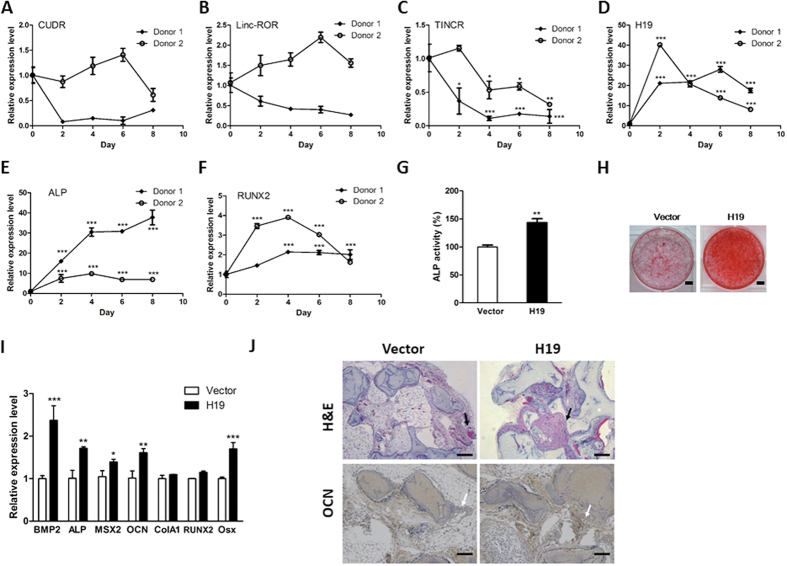
Overexpression of H19 promoted osteogenesis. (**A**–**F**) The hMSCs isolated from two independent donors’ bone marrow were initiated by osteogenic medium and harvested at indicated time points. The expression levels of several candidate lncRNAs, including CUDR (**A**), Linc-ROR (**B**), TINCR (**C**), H19 (**D**), ALP (**E**) and RUNX2 (**F**) were monitored by qRT-PCR. (**G**) The osteogenesis of H19-overexpressing hMSCs was initiated by osteogenic medium. At day 10 post-induction, the ALP activity was measured and ectopic expression of H19 enhanced ALP activity. (**H**) The osteoblast differentiation of H19-overexpressing hMSCs was started by osteogenic medium. At day 20 post-induction, the calcified nodules were visualized by Alizarin Red stainning and it was found that overexpression of H19 promoted hMSC osteogenesis. Scale bars: 1cm. (**I**) The RNA levels of osteogenesis-related marker genes were evaluated by qRT-PCR and ectopic expression of H19 upregulated the expression serial osteogenic maker genes. (**J**) H19-overexpressing hMSCs and corresponding control cells were implanted subcutaneously into the dorsal surfaces of nude mice. At 8 weeks post-implantation, the transplants were collected for histological analysis. Representative images of H&E staining and immunohistochemical staining of OCN were captured. The new bone matrix was indicated by black arrow and OCN proteins were highlighted by white arrow. Scale bars: 100 μm. The results of *in vivo* bone formation assay showed that H19 significnatly enhanced osteogenesis. (n = 3; *P < 0.05; **P < 0.01; ***P < 0.001).

To identify the potential lncRNAs involved in osteogenesis, five previously reported lncRNAs, namely CUDR, Linc-ROR, TINCR, H19 and Linc-MD1, were chosen and subjected to qRT-PCR analysis to evaluate their expression patterns during osteoblast differentiation. In this study, we utilized osteogenic differentiation medium to initiate the osteoblast differentiation in hMSCs from two independent donors. The RNA samples were collected and examined at indicated time points. According to the RT-PCR results, CUDR displayed opposite expression patterns in two primary hMSCs from different donors (Fig. 1A). Similar results were achieved in the RT-PCR analysis of Linc-ROR (Fig. 1B). And the lncRNA Linc-MD1 was undetectable in our study (data not shown). Intriguingly, two lncRNAs TINCR and H19 exhibited remarkable expression alteration during osteogenesis (Fig. 1C,D). We also examined two osteogenic marker genes ALP (Fig. 1E) and RUNX2 (Fig. 1F), both of which displayed significant increase during osteogenesis. In terms of the dramatic changes during osteogenesis, H19 is of great interest due to its elusive biological importance in cell differentiation. To validate the expression pattern of H19, we also took advantage of semi-qRT-PCR and confirmed that H19 was significantly upregulated during osteogenesis (Supplementary Fig. S1A). Further studies also demonstrated that osteogenesis inducers TGF-β1, BMP2 and BMP4 could increase the expression of H19, suggesting that H19 may provoke osteoblast differentiation (Supplementary Fig. S1B–D).

We next generated stable H19-overexpressing hMSCs by retroviral system and the establishment of stable cell line was verified by RT-PCR (Supplementary Fig. S2A,B). Then, the osteoblast differentiation was initiated in the stable cell lines expressing H19. Based on the results of ALP assay and Alizarin Red S staining assays, it was shown that H19 significantly promoted hMSCs osteogenesis when compared with control group (Fig. 1G,H). We also illustrated that overexpression of H19 could activate the expression of several osteogenic marker genes such as BMP2, OCN and MSX2 (Fig. 1I). Furthermore, the *in vivo* bone formation capacity of H19-overexpressing hMSCs was also assessed and H19-overexpressing hMSCs displayed enhanced ectopic bone formation in our study (Fig. 1J).

### H19 functions as miRNA sponge for miR-141 and miR-22

**Figure 2:**
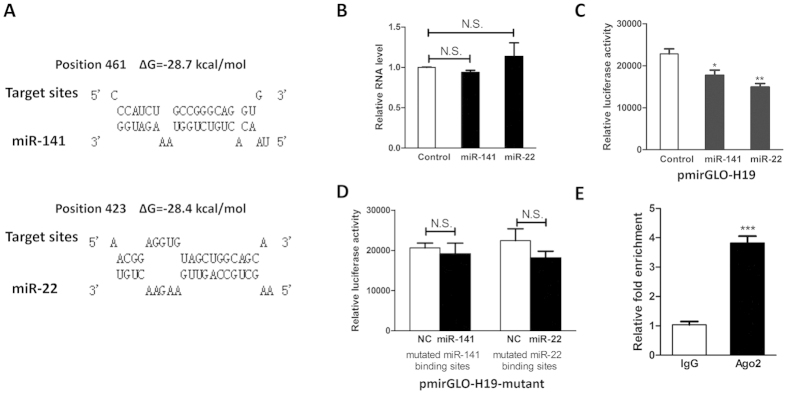
H19 was a *bona fide* target for miR-141 and miR-22. (**A**) Schematic diagrams of the mutual interplays between miRNA and H19. The positions of miRNA binding sites and calculated ΔG values are showed on the top (kcal/mol). (**B**) qRT-PCR results showed the relative RNA level of H19 after ectopic expression of miR-141 and miR-22. Neither miR-141 nor miR-22 affected the RNA level of H19. (**C**) HEK293 cells were transfected with miRNA mimics combined with luciferase reporter harboring H19 gene. The effects of miR-141 and miR-22 on the luciferase activity were measured by luciferase reporter assays. MiR-141 and miR-22 could suppress the luciferase activity. (**D**) The miR-141 and miR-22 binding sites were mutated and the mutated luciferase reporters were co-transfected with corresponding miRNAs. The mutations on binding sites abolished the previously suppressive effect. (**E**) H19 RNA level in the immunoprecipitates were measured by qRT-PCR. H19 RNA was enriched in the Ago2-coated beads. (n = 3; *P < 0.05; **P < 0.01; ***P < 0.001).

Recent studies demonstrated that H19 directly interacted with miRNA ribonucleoprotein complexes and acted as a decoy for let-7, resulting in the de-repression of several protein-coding genes targeted by let-7^17^. Similar results were achieved by our group and we successfully presented that, aside from let-7, H19 could also act as a natural molecular sponge to hijack miR-138 and miR-200a^18^. These findings promoted us to investigate whether other miRNAs can target H19. We first took advantage of a bioinformatic tool to screen out the candidate miRNAs targeting H19^19^. Subsequent bioinformatic analysis identified putative binding sites for miR-141 and miR-22 in human H19 (Fig. 2A). However, neither miR-141 nor miR-22 affected the RNA level of H19 (Fig. 2B). Given that miRNA may target candidate genes by triggering transcriptional inhibition rather than RNA degradation, we suspected that certain miRNAs may influence the function of H19 without altering their RNA level. To validate this hypothesis, we next generated a luciferase reporter by placing H19 full length sequence in the 3′UTR region of firefly luciferase gene, which acted as an indicator in our study. Based on the luciferase assay results, we showed that miR-141 and miR-22 significantly repressed the luciferase activity of luciferase reporter containing H19 sequence (Fig. 2C). Then, we mutated these binding sites by site-directed mutagenesis and demonstrated that mutations on the binding sites for miR-141 and miR-22 successfully abolished the previous suppressive effect (Fig. 2D), suggesting that miR-141 and miR-22 did target H19 by inducing transcriptional suppression rather than directly degrading H19 RNA transcript.

In the past decade, it is well characterized that miRNA exerts its function by associating to Ago2, a major constituent of the RNA-induced silencing complex. To assess whether H19 physically interacts with RISC complex and hence attenuates the function of miRNAs, RNA immunoprecipitation assay was conducted by using specific antibody against Ago2 protein. According to the results from RIP assay, H19 was found to be preferentially enriched in Ago2-coating beads compared with control group (Fig. 2E). Collectively, our results pinpointed a functional role of H19 as a natural miRNA decoy for miR-141 and miR-22.

### MiR-141 and miR-22 are negative modulator of osteogenesis

**Figure 3:**
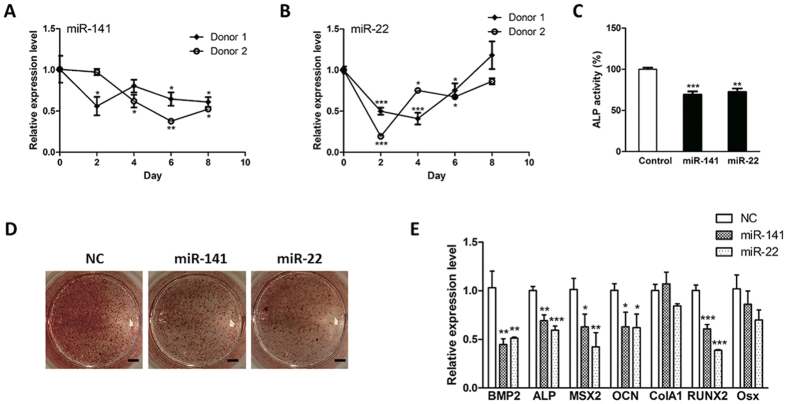
Overexpression of miR-141 and miR-22 suppressed osteoblast differentiation. (**A,B**) The expression levels of miR-141 (**A**) and miR-22 (**B**) were determined by qRT-PCR. These two miRNAs were downregulated during osteogenesis. (**C**) The hMSCs were tranfected with miR-141 or miR-22 mimics. The hMSC osteogenesis was initiated by osteogenic medium. At day 10 post-induction, the ALP activity was measured and ectopic expression of both miR-141 and miR-22 impaired ALP activity. (**D**) The osteoblast differentiation of miR-141- or miR-22-overexpressing hMSCs was started by osteogenic medium. At day 20 post-induction, the calcified nodules were visualized by Alizarin Red stainning and it was showed that overexpression of miR-141 or miR-22 inhibited hMSC osteogenesis. Scale bars: 1 cm. (**E**) The RNA levels of osteogenesis-related marker genes were evaluated by qRT-PCR and ectopic expression of miR-141 or miR-22 downregulated the expression serial osteogenic maker genes. (n = 3; *P < 0.05; **P < 0.01; ***P < 0.001).

To elucidate the biological effect of miR-141 and miR-22, we examined the expression profiles of these two miRNAs during osteogenesis and both miR-141 and miR-22 displayed significant downregulation (Fig. 3A,B). We next assessed the effect of miR-141 and miR-22 in modulating osteogenesis by periodically transfecting hMSCs with miRNA mimics. It was shown that, when compared to control group, these two miRNAs could suppress the ALP activity, which is widely used as a putative osteogenesis marker (Fig. 3C). Further Alizarin Red S staining confirmed that miR-141 and miR-22 significantly alleviated the formation of calcium nodules during osteoblast differentiation (Fig. 3D). Thereafter, a variety of osteogenesis-related marker genes were examined by RT-PCR and it was displayed that miR-141 and miR-22 downregulated most of the osteogenesis marker genes (Fig. 3E), suggesting that these two miRNAs were potential negative regulators of osteoblast differentiation.

### Beta-catenin is a common target for miR-141 and miR-22

**Figure 4:**
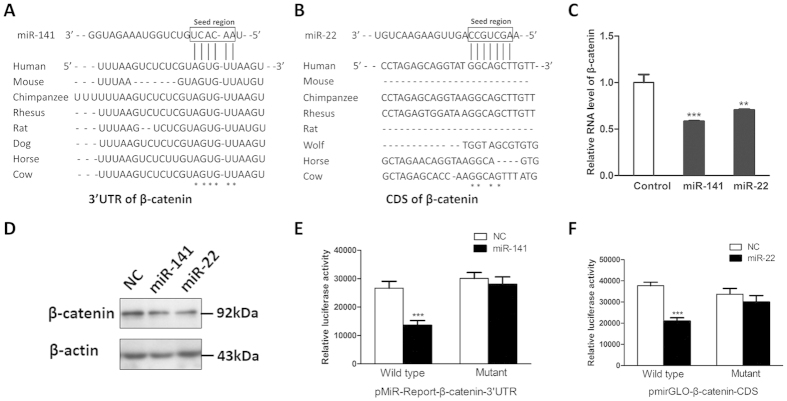
β-catenin was a *bona fide* target for miR-141 and miR-22. (**A**) The 3′-UTR region of β-catenin harbors a putative miR-141 binding site, which is highly conserved across multiple species. (**B**) The protein-coding region of β-catenin contains a potential miR-22 binding site, which is conserved across a number of species. (**C**) Overexpression of miR-141 or miR-22 dramatically reduced the mRNA levels of β-catenin in hMSCs. (**D**) Overexpression of miR-141 or miR-22 decreased the protein levels of β-catenin in hMSCs. (**E**) The interaction between miR-141 and 3′UTR region of β-catenin was confirmed by luciferase reporter assays in HEK293 cells. (**F**) The interaction between miR-22 and protein-coding region of β-catenin was verfied by luciferase reporter assays in HEK293 cells. (n = 3; *P < *0.05*; **P < *0.01*; ***P < *0.001*).

In terms of the biological importance of miR-141 and miR-22, we were interested in the candidate protein-coding genes targeted by these two miRNAs. According to bioinformatics analysis, we screened out that β-catenin might be a common target for miR-141 as well as miR-22, and β-catenin is of great interest to us due to its fundamental role in osteogenesis^20^. Interestingly, in previous study, β-catenin was characterized as a known target of miR-200, which shares similar seed region with miR-141^21^. Based on sequence alignments, the binding sites for miR-141 and miR-22 are highly conserved across diverse mammalian species (Fig. 4A,B). But, different from miR-141, the binding sites of miR-22 are located in the coding region rather than UTR region. By transfecting hMSCs with miRNA mimics, we demonstrated both miR-141 and miR-22 significantly downregulated the expression of β-catenin at RNA and protein levels (Fig. 4C,D). Furthermore, we also transfected cells with miR-141 and miR-22 inhibitors and it was showed that inhibition of miR-141 and miR-22 by their respective inhibitors upregulated the expression level of β-catenin (Supplementary Fig. S3). In order to validate whether β-catenin is a *bona fide* target for miR-141 and miR-22, we inserted the target sites into the 3′UTR locus of firefly luciferase gene. It was shown that these two miRNAs dramatically suppressed the luciferase activity of the β-catenin 3′UTR locus when compared with that of the control groups and mutations on these miRNA binding sites successfully abolished the suppressive effects (Fig. 4E,F). Taken together, according to our results, miR-141 and miR-22 were identified as two novel regulators of β-catenin.

### H19 activates Wnt/beta-catenin pathway

**Figure 5:**
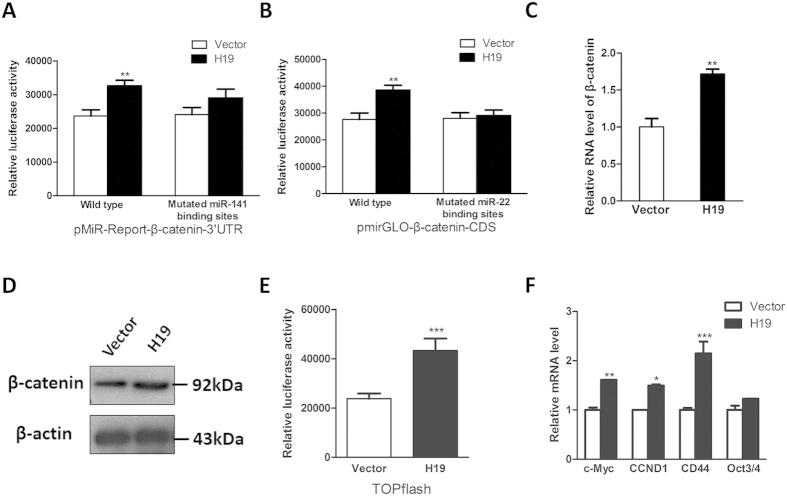
Upregulation of H19 increased the expression of β-catenin and activated Wnt/β-catenin pathway. (**A**) Luciferase reporter assays were utilized to monitor the interaction between H19 and luciferase reporters containing the 3′UTR region of β-catenin. Overexpression of H19 upregulated the luciferase activity of wild type luciferase reporter while mutation on miR-141 binding sites counteracted this upregulation effect. (**B**) Luciferase reporter assays were used to evaluate the interplay between H19 and luciferase reporters containing the partial coding region of β-catenin. Ectopic expression of H19 upregulated the luciferase activity of wild type luciferase reporter while mutation on miR-22 binding sites eliminated this upregulation effect. (**C,D**) Upregulation of H19 increased the RNA (**C**) and protein (**D**) levels of β-catenin. (**E**) Overexpression of H19 enhanced the TOPflash luciferase activity. (**F**) Upregulation of H19 activated some downstream transcriptional targets of β-catenin. (n = 3; *P < 0.05; **P < 0.01; ***P < 0.001).

Recently, emerging evidence suggests that lncRNA might modulate other mRNA transcripts by competing for shared endogenous miRNAs^22^. These findings promoted us to posit that H19 could regulate β-catenin expression in this manner. First, we examined the effect of H19 in modulating miR-141 and miR-22 binding sites within β-catenin RNA transcript. We transfected H19 expression vector and luciferase reporters harboring miRNA binding sites into HEK293 cells and showed that H19 significantly upregulated the luciferase activity of luciferase reporters (Fig. 5A,B). However, the upregulated effects were counteracted by mutations on miRNA binding sites (Fig. 5A,B), suggesting that H19 may act as an miRNA sponge and hence modulate β-catenin expression. Then, we showed that H19 potentiated the expression of β-catenin in both the RNA and protein levels (Fig. 5C,D). Consistent with these results, H19 was found to activate the luciferase activity of Wnt signaling reporter TOPFlash, which contains three binding sites for TCF and β-catenin (Fig. 5E). Furthermore, the RNA levels of several downstream transcriptional targets such as c-Myc and CCND1 were increased after ectopic expression of H19 (Fig. 5F). On the other hand, the effects of the loss of function of H19 were also examined in our study and we showed that downregulation of H19 by siRNA could significantly suppress the Wnt/β-catenin pathway (Supplementary Fig. S4), indicating that H19 plays a role in mediating the activation of Wnt/β-catenin pathway.

### MiR-675-5p alleviates osteoblast differentiation

**Figure 6:**
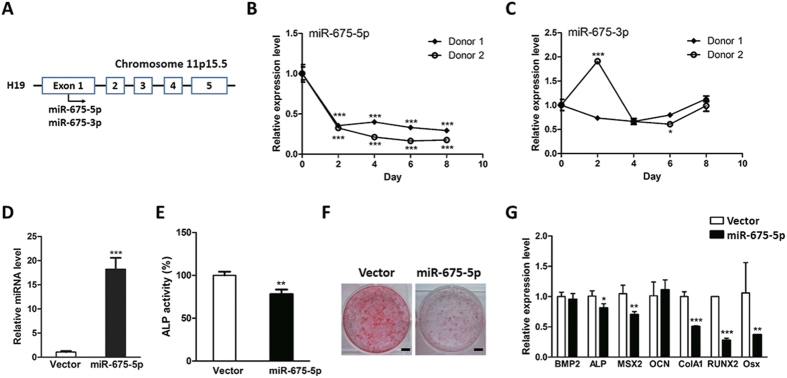
Overexpression of miR-675-5p suppressed osteoblast differentiation. (**A**) The schematic representation of the H19 transcript and its encoded miR-675. (**B,C**) The expression levels of miR-675-5p (**B**) and miR-675-3p (**C**) were determined by qRT-PCR. (**D**) The hMSCs were tranfected with miR-675-5p mimics and the effects of transient transfection were confirmed by qRT-PCR. (**E**) The osteogenesis of miR-675-5p-overexpressing hMSCs was initiated by osteogenic medium. At day 10 post-induction, the ALP activity was measured and ectopic expression of miR-675-5p impaired ALP activity. (**F**) The osteoblast differentiation of miR-675-5p-overexpressing hMSCs was started by osteogenic medium. At day 20 post-induction, the calcified nodules were visualized by Alizarin Red stainning and it was showed that overexpression of miR-675-5p suppressed hMSC osteogenesis. Scale bars: 1 cm. (**G**) The RNA levels of osteogenesis-related marker genes were evaluated by qRT-PCR and ectopic expression of miR-675-5p downregulated the expression serial osteogenic maker genes. (n = 3; *P < 0.05; **P < 0.01; ***P < 0.001).

It was reported that some miRNAs are transcribed from the intragenic loci even though the region are producing ncRNA. Indeed, exon 1 of H19 harbors a primary miRNA sequence, which generates miR-675-3p and miR-675-5p (Fig. 6A)^23^. Intriguingly, the stem loop region within this locus is highly conserved across various species. To characterize whether these two miRNAs play a role in H19-mediated osteogenesis, we evaluated their expression during osteogenesis. However, unexpectedly, neither miR-675-5p nor miR-675-3p exhibited coordinated pattern with H19 (Fig. 6B,C). Furthermore, unlike H19’s upregulation during osteogenesis, miR-675-5p displayed gradually downregulation during the differentiation towards osteoblast (Fig. 6B). We then periodically transfected hMSCs with miR-675-5p mimics and its upregulation were confirmed by RT-PCR (Fig. 6D). The osteoblast differentiation was then initiated and we showed that ectopic expression of miR-675-5p could suppress the hMSCs osteogenesis (Fig. 6E,F). Moreover, a variety of osteogenesis-promoting genes were found to be downregulated by miR-675-5p (Fig. 6G), suggesting miR-675-5p is a negative regulator of osteogenesis.

As the number of osteoblasts is important for maintaining the bone homeostasis, we would like to know whether H19 or miR-675-5p might affect osteogenesis *via* regulating cell proliferation. Thus, we also studied the effect of H19 and miR-675-5p in modulating cell cycle progression. However, in the serial proliferation-related assays, no significant alteration was observed in cell proliferation assays or flow cytometry assays (Supplementary Fig. S5), suggesting that neither H19 nor miR-675-5p exerted their function in osteogenesis through modulating cell proliferation.

### H19 is negatively regulated by miR-675-5p

**Figure 7:**
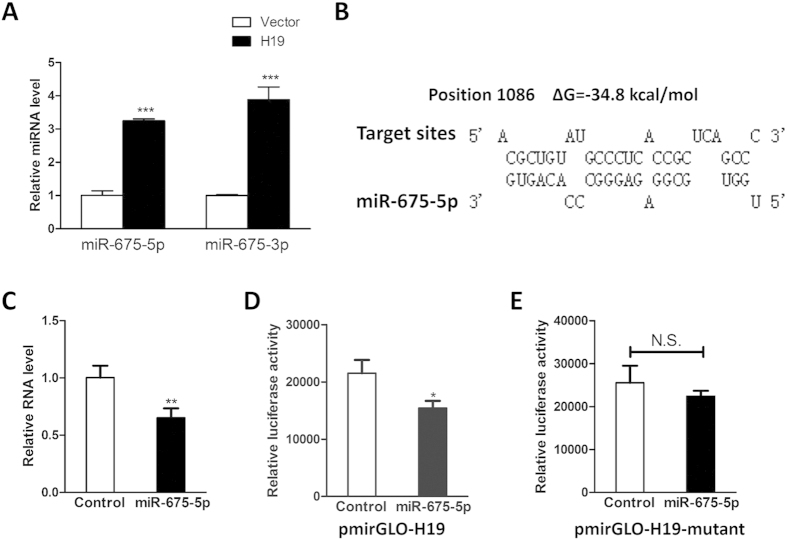
Overexpression of H19 gave rise to miR-675-5p and miR-675-3p while miR-675-5p downregulated the expression of H19. (**A**) The expression levels of miR-675-5p and miR-675-3p were determined by qRT-PCR in the H19-overexpressing hMSCs. Ectopic expression of H19 significantly increased the expression of miR-675-5p and miR-675-3p. (**B**) Schematic diagrams of the mutual interplays between miR-675-5p and H19. The positions of miR-675-5p binding sites and calculated ΔG values are showed on the top (kcal/mol). (**C**) qRT-PCR results showed the relative RNA level of H19 after ectopic expression of miR-675-5p. Overexpression of miR-675-5p downregulated the RNA level of H19. (**D**) HEK293 cells were transfected with miR-675-5p mimics combined with luciferase reporter harboring H19 gene. The effects of miR-675-5p on the luciferase activity were measured by luciferase reporter assays and overexpression of miR-675-5p could suppress the luciferase activity. (**E**) The miR-675-5p binding sites within luciferase reporter plasmid were mutated. HEK293 cells were transfected with miR-675-5p mimics combined with mutated luciferase reporter harboring H19 gene. The mutatation abolished the suppressive effect. (n = 3; *P < 0.05; **P < 0.01; ***P < 0.001).

Given that H19 is a developmental reservoir of miR-675-5p and miR-675-3p, to shed light on the molecular mechanism on how miR-675-5p modulates osteogenesis, we next investigated the interplay between miR-675-5p and H19. In accordance with previous studies^16^,^23^, overexpression of H19 increased the expression of miR-675-5p and miR-675-3p (Fig. 7A). In terms of the opposite expression patterns during osteogenesis (Figs 1D and 6B), we suspected that a regulatory feedback loop may exist between miR-675-5p and its host gene H19. We then took advantage of bioinformatic tools and identified putative miR-675-5p binding site within H19 RNA transcripts (Fig. 7B). The RT-PCR result validated that overexpression of miR-675-5p significantly downregulated the RNA level of H19 (Fig. 7C). The luciferase assays showed that ectopic expression of miR-675-5p suppressed the luciferase activity of luciferase reporter harboring full length H19, while mutating this binding sites successfully counteracted the suppressive effect (Fig. 7D,E), indicating that miR-675-5p may target H19 through this binding site. Taken together, our results showed that ectopic expression of H19 gave rise to miR-675-5p and miR-675-3p while miR-675-5p could target H19, which forms a negative regulatory feedback loop. These results might partially account for why miR-675-5p displayed an opposite expression pattern and its functional role in osteogenesis when compared with that of its host gene H19.

## Discussion

As an emerging worldwide epidemic, osteoporosis affects a large number of people all over the world, especially the elderly individuals and postmenopausal women. Osteoporosis is a disease characterized by decreased bone density and subdued strength, which eventually results in fragility fractures. Based on numerous genetic studies and genome-wide association investigations, it is well-defined that canonical Wnt/β-catenin pathway is a vital positive modulator of bone homeostasis^20^. In terms of the biological importance of Wnt/β-catenin pathway in osteogenesis, this signaling pathway is a promising target for developing therapeutic intervention to attenuate loss of bone mass and improve public health. As a matter of fact, another group previously demonstrated the H19 activated Wnt/β-catenin pathway by interacting with the RNA binding protein Enhancer of zeste homolog 2 (EZH2), leading to enhanced bladder cancer metastasis^24^. However, the missing link between H19 and Wnt/β-catenin pathway remains largely unknown.

Recently, the ceRNA hypothesis proposed that lncRNAs communicated with other protein-coding RNA transcripts through sharing common miRNA binding sites^22^. In accordance with the ceRNA hypothesis, several innovative biochemical approaches have successfully identified the potential interaction maps between miRNA and lncRNA^25^,^26^ pinpointing an important role of miRNAs in mediating lncRNA function. Furthermore, in the past decade, lots of lncRNAs including H19 have been implied to act as an miRNA sponge and thus modulate a variety of cellular events^27^,^28^,^29^. In our current study, we characterized the expression profiles as well as the function of H19 during osteogenesis. Furthermore, based on the ceRNA hypothesis, revealed a previously unknown mechanism that H19 potentiated Wnt/β-catenin pathway by serving as a molecular sponge for miR-141 and miR-22 (Supplementary Fig. S6), leading to the promotion of osteoblast differentiation. Meanwhile, we also identified a self-regulatory feedback loop between H19 and its encoding miR-675-5p, which shed light on the complicated molecular interplay concerning H19 and miRNAs.

Given that H19 is a developmental reservoir of miR-675, our study also raised an interesting question about why H19 did not display coordinated expression patterns with miR-675-5p. However, our observation is not unique since inconsistent expression between H19 and miR-675 was shown during placental growth^16^. Moreover, the excision of miR-675 from exon 1 of H19 is dynamically modulated by the RNA binding protein HuR, which strongly attenuates the processing of miR-675-5p and miR-675-3p^16^. But, unfortunately, less is known about the expression patterns of RNA binding proteins during osteogenesis.

Previous studies demonstrated that miR-675-5p precursor is derived from the exon 1 of H19 RNA transcript in the nucleus, the processing of which is mediated by Drosha/DGCR8. In the nucleus, the 5′ end cap of H19 transcript is removed by the Drosha/DGCR8 or spliceosome, which results in decreased RNA stability and cytoplasmic transportation of H19 transcript. During hMSCs osteoblast differentiation, it is possible that some RNA binding proteins such as HuR bind to the H19 RNA transcript and thus inhibit the processing of miR-675. This inhibition may eventually trigger the opposite expression patterns between H19 and miR-675. Taken together, these findings suggest that some potential RNA binding proteins like HuR may play a role in regulating osteogenesis and further investigations are required for elucidating the functional role of RNA binding proteins in osteoblast differentiation.

Plenty of recent studies indicated that miRNA orchestrates a broad spectrum of cellular processes including bone homeostasis and skeletal development. Meanwhile, it is widely believed that miRNAs has emerged as a fundamental modulator of osteogenesis-related signaling pathways^30^. For instance, consistent with our results, miR-141 were found to be upregulated in response to BMP2 treatment in murine MC3T3-E1 cells and miR-141 negatively modulated osteogenesis by targeting DLX5, an activator of Runx2 and Osterix^31^. However, as for miR-22, opposite expression profiles and contradictive function was reported by another group. They revealed that miR-22 was significantly increased during osteogenic differentiation and ectopic expression of miR-22 promoted the osteogenesis of human adipose tissue-derived mesenchymal stem cells (hADMSCs)^32^. Nevertheless, contradictive results have been reported on other miRNAs in previous studies. For example, miR-27, which is negatively regulated by Runx2 and hence downregulated during osteogenesis, attenuated osteoblast differentiation by targeting SATB2 in murine preosteoblast MC3T3-E1 cells^33^. On the contrary, in another study, miR-27 was found to potentiate osteoblast differentiation in hFOB1.19 cells^34^. Of course, some acceptable explanations could account for this dilemma such as the diverse cell types or culture conditions used in their independent systems. However, these findings also indicated the sophisticated regulatory mechanisms in osteoblast differentiation.

In the present study, we revealed a previously uncharacterized role of H19 in regulating osteogenesis. And we also demonstrated that H19 functioned as an miRNA sponge to attenuate the endogenous function of miR-141 and miR-22, both of which negatively modulate β-catenin expression. Hence, by competing for these shared miRNAs withβ-catenin, H19 enhanced osteogenesis by direct activation of Wnt/β-catenin pathway.

## Materials and Methods

### Cell culture and induction of osteoblast differentiation

HEK293 cells were maintained in Dulbecco’s modified Eagle medium (DMEM) supplemented with 10% fetal bovine serum, 100 U/ml penicillin, and 100 μg/ml streptomycin. Based on the cell surface makers, the hMSCs were identified and isolated by flow cytometry as previously described^35^,^36^. The informed consent was obtained from all subjects and this study is approved by the Medical Ethical Committee of The Chinese University of Hong Kong. Briefly, after aspiration from healthy donors’ bone marrow, hMSCs were isolated and identified by cell surface markers like CD3-PE, CD16-FITC, CD19-FITC, CD33-FITC, CD34-PE, CD38-FITC, CD45-FITC, CD133-PE. Thereafter, the hMSCs were cultured in Minimum Essential Media Alpha, supplemented with 10% fetal bovine serum, 100 U/ml penicillin, and 100 μg/ml streptomycin. The hMSCs were seeded into a 12-well plate at a density of 5 × 10^5^ cells per well. Then the osteoblast differentiation was initiated when the culture medium was supplemented with 10^−8^ M dexamethasone (Sigma-Aldrich,USA), 50 μg/ml ascorbic acid 2-phosphate (Sigma-Aldrich,USA), and 10 mM glycerol 2-phosphate (Sigma-Aldrich,USA). The differentiation medium was replaced by fresh medium every 3 days.

### Establishment of stable cell lines

The H19 overexpression stable hMSCs were generated using retrovirus-mediated gene delivery method as previously described^37^. To produce retrovirus for infection, 3 μg pBABE-H19 vector or corresponding control empty vector were co-transfected with an equal amount of viral packaging vector pCL-Ampho into HEK293 cells in 100 mm culture dish by using Lipofectamine 2000 transfection reagent (Invitrogen, USA). After transfection, the supernatant containing retrovirus particles were collected and subsequently filtered by 0.45 μm pore size nitrocellulose membranes (Millipore, USA). The hMSCs were infected by the retroviral particles together with 8 μg/mL Hexadimethrine bromide (Sigma-Aldrich, USA). Then, retrovirus infected hMSCs were selected by puromycin (Sigma-Aldrich, USA) in the concentration of 0.5 μg/mL. After antibiotics selection for around 7 days, some cultured cells were collected and the overexpression of H19 was confirmed by qRT-PCR.

### RNA extraction and qRT-PCR

Total RNAs from hMSCs were isolated by TRIZOL Reagent (Invitrogen, USA) following the manufacturer’s instructions. After RNA extraction, RNA samples were reversely transcribed by High Capacity cDNA Reverse Transcription Kit (Applied Biosystems, USA). As for the detection of miRNA, the NCode miRNA First-Strand cDNA Synthesis kit (Invitrogen, USA) was applied in our study. Then, the Fast start Universal SYBR Green Master (Roche, USA) was used for the quantitative Real Time–PCR (qRT-PCR). The primer sequences are available in Supplementary Table 1. The relative expression levels of target genes were normalized to housekeeping gene RPLPO and analyzed by 2^−ΔΔCT^ method.

### Western blot

All the cell lysates were harvested by RIPA buffer (Sigma-Aldrich, USA) with Complete Protease Inhibitor Cocktail (Roche, USA). The cellular proteins were separated by 10% SDS-PAGE and subsequently transferred to PVDF membrane (Millipore, USA). The membranes were probed with the following antibodies: β-catenin (Cell Signaling Technology, USA) and β-actin (Sigma, USA). The results were visualized in the X-Ray film by Kodak film developer (Fujifilm, Japan).

### Cell transfection and plasmid construction

All the miRNA mimics were purchased from GenePharma Company (Shanghai, China). The miRNA mimics and DNA plasmid were transfected by using transfection reagent Lipofectamine 2000 (Invitrogen, USA). The pmirGLO-H19 luciferase plasmid were constructed in previous study. The mutated miR-141 and miR-22 binding sites within H19 luciferase reporter vector were generated by site-directed mutagenesis method.

### Measurement of ALP activity and Alizarin Red staining

The hMSCs were seeded into 24 well plates at a density of 2 × 10^5^ cells per well and osteoblast differentiation was induced when cells reach 100% confluence. Cell lysates were harvested and ALP activity was evaluated and analyzed following the manufacturer’s instructions (ALP diagnostics kit; Sigma-Aldrich). For the alizarin red staining, the hMSCs were fixed in 4% Paraformaldehyde and then hMSCs were stained with 2% Alizarin Red staining solution. After Alizarin Red staining, the orange and red spots were regarded as calcified nodules.

### Bone formation assay and IHC

The H19-overexpressing hMSCs and corresponding control cells were loaded onto sterilized Skelite resorbable bone graft substitute (Kingston, ON, Canada) and subsequently incubated at 37 °C for 3 hours, allowing hMSCs to attach to the graft. The nude mice were anaesthetized and the grafts with the hMSCs were subcutaneously implanted into the dorsal sides of nude mice. At 8 weeks post-implantation, the xenografts were harvested and subjected to histological analysis. The sections from xenografts were subjected to Hematoxylin and Eosin (H&E) staining as well as immunohistochemical staining targeting OCN. This study was conducted in accordance with the guidelines and approved by the Medical Ethical Committee of The Chinese University of Hong Kong.

### Luciferase assay

Luciferase assays were conducted based on a previously published protocol^38^,^39^. Briefly, HEK293 cells were seeded into a 24 well plate, and then transfected with luciferase plasmids as well as miRNA mimics. The pRSV-β-Galactosidase vector was co-transfected into HEK293 cells as an internal control for normalization. The firefly luciferase activity was monitored through the Luciferase Reporter Assay System (Promega, USA). The O-nitrophenyl-β-galactoside (ONPG) colorimetric assays were conducted to measure the β-Galactosidase activity and the relative luciferase activity was calculated after normalization with the β-Galactosidase activity.

### RNA immunoprecipitation

RNA immunoprecipitation assays were conducted as previously reported with slight modification^40^. H19-overexprssing hMSCs were rinsed with PBS and subsequently fixed by 1% formaldehyde. The cell pellets were harvested by centrifugation and re-suspended in the NP-40 lysis buffer (Sigma-Aldrich, USA) with 1mM PMSF (Sigma-Aldrich, USA), 1mM DTT (Invitrogen, USA), 1% Protease Inhibitor Cocktail (Sigma-Aldrich, USA) as well as 200 U/ml RNase Inhibitor (Life Technologies, USA). The supernatant from cell lysates was harvested by centrifugation at the speed of 14000 rpm. To produce antibody-coated beads, Protein G Sepharose 4 Fast Flow bead slurry (GE Healthcare, USA) was washed with cold NT2 buffer (50 mM Tris-HCl, 0.5% NP-40, 150 mM NaCl, 1 mM MgCl_2_) and subsequently incubated with antibody against Ago2 (Abcam, UK) or corresponding negative control IgG (Sigma-Aldrich, USA). To precipitate the RNA bound to Ago2, the supernatant was co-incubated with the antibody-coated beads overnight. To remove the non-specific binding, the Sepharose beads were rinsed with cold NT2 buffer and then incubated with 10 mg/ml proteinase K (Sigma-Aldrich, USA). The RNA transcripts associated with Ago2 antibody were isolated by TRIzol reagent (Invitrogen, USA) according to the supplier’s instructions.

### Statistical analysis

Experimental data are expressed as Mean ± SD. Statistical comparisons between two groups were analyzed by using two-tail Student’s T-test. The results were considered to be statistically significant when *P* < 0.05.

## Additional Information

**How to cite this article**: Liang, W.-C. *et al.* H19 activates Wnt signaling and promotes osteoblast differentiation by functioning as a competing endogenous RNA. *Sci. Rep.* **6**, 20121; doi: 10.1038/srep20121 (2016).

## Supplementary Material

Supplementary Information
