# Supplementary material for: H19 activates Wnt signaling and promotes osteoblast differentiation by functioning as a competing endogenous RNA
Source: Sci Rep. 2016 Feb 8;6:20121. doi: 10.1038/srep20121 (PMC4745008; doi:10.1038/srep20121)
Supplement: Supplementary Information [file srep20121-s1.pdf]

H19 activates Wnt signaling and promotes osteoblast differentiation by functioning as a competing endogenous RNA

Wei-Cheng Liang[1,2,#], Wei-Ming Fu[3,#], Yu-Bing Wang[1,2], Yu-Xin Sun[4], Liang-Liang Xu[4,5], Cheuk-Wa Wong[1,2], Kai-Ming Chan[4,6], Gang Li[4,5,6], Mary Miu-Yee Waye[1,2*], Jin-Fang Zhang[4,5,6,7**]

[1]School of Biomedical Sciences, The Chinese University of Hong Kong, Shatin, New Territories, Hong Kong, P.R. China

[2]Croucher Laboratory for Human Genomics, The Chinese University of Hong Kong, Shatin, New Territories, Hong Kong, P.R. China

[3]Guangzhou Institute of Advanced Technology, Chinese Academy of Sciences, Guangzhou, 510000, P.R. China

[4]Department of Orthopaedics & Traumatology, The Chinese University of Hong Kong, Prince of Wales Hospital, Shatin, Hong Kong, P.R. China

[5] Shenzhen Research Institute, The Chinese University of Hong Kong, Shenzhen, P.R. China.

[6]Stem Cells and Regenerative Medicine Laboratory, Lui Che Woo Institute of Innovative Medicine, Li Ka Shing Institute of Health Sciences, The Chinese University of Hong Kong, Prince of Wales Hospital, Shatin, Hong Kong, P.R. China

[7]School of medicine, South China Unversity of Technlogy, Guangzhou, 510000, P.R. China

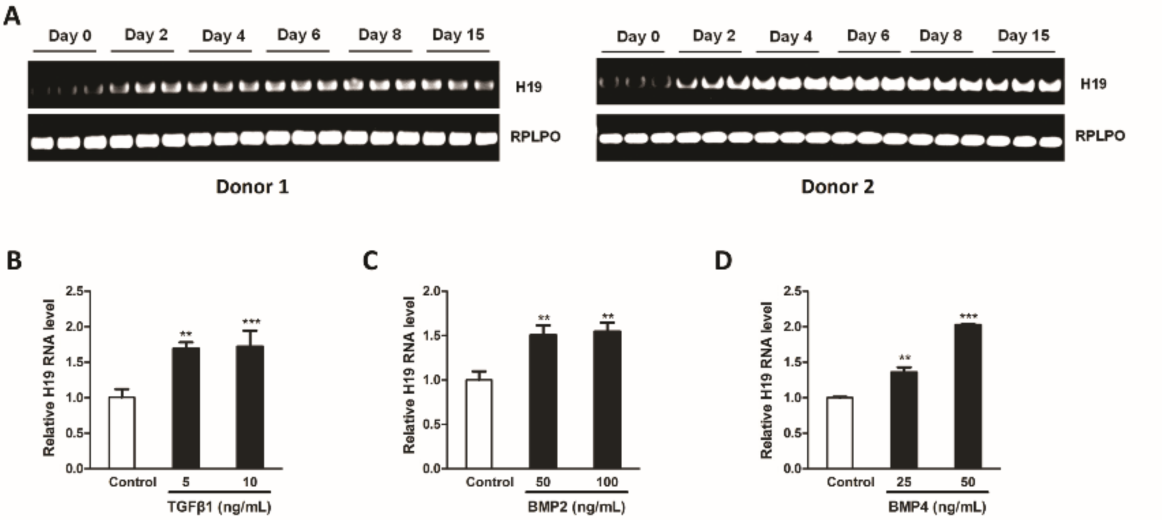

Supplementary Figure 1. H19 was increased during osteoblast differentiation. (A) H19 was significantly upregulated during osteoblast differentiation from hMSCs to osteoblast. Similar expression profiles were identified in two hMSCs from independent donors. (B-D) H19 was increased in response to several osteogenesis-related growth factors, namely TGF-β1, BMP2 and BMP4.

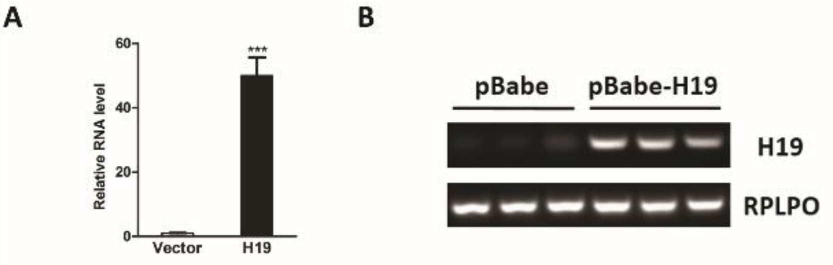

Supplementary Figure 2. RT-PCR and semi- RT-PCR confirmed the upregulation of H19 in the stable hMSC cell line.

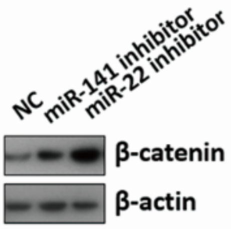

Supplementary Figure 3. Inhibition of miR-141 and miR-22 by their respective inhibitors upregulated the expression level of β-catenin.

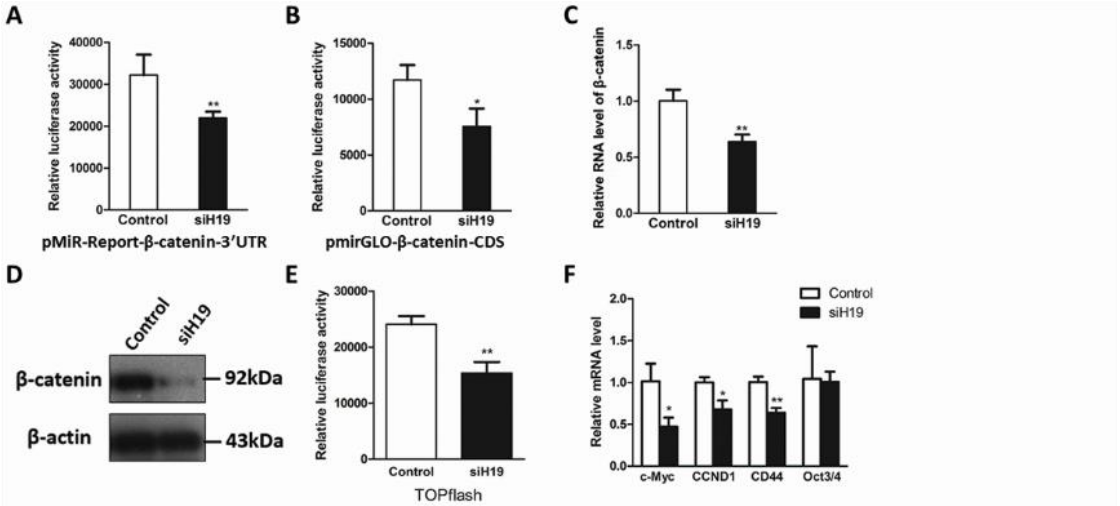

Supplementary Figure 4 . Downregulation of H19 decreased the expression of β-catenin and inactivated Wnt/β-catenin pathway. (A&B) Downregulation of H19 by siRNA significantly downregulated the luciferase activity of two luciferase reporters containing different regions of β-catenin. (C&D) Downregulation of H19 decreased the RNA (C) and protein (D) levels of β-catenin. (E) Silencing of H19 impaired the TOPflash luciferase activity. (F) Silencing of H19 repressed some transcriptional targets of β-catenin.

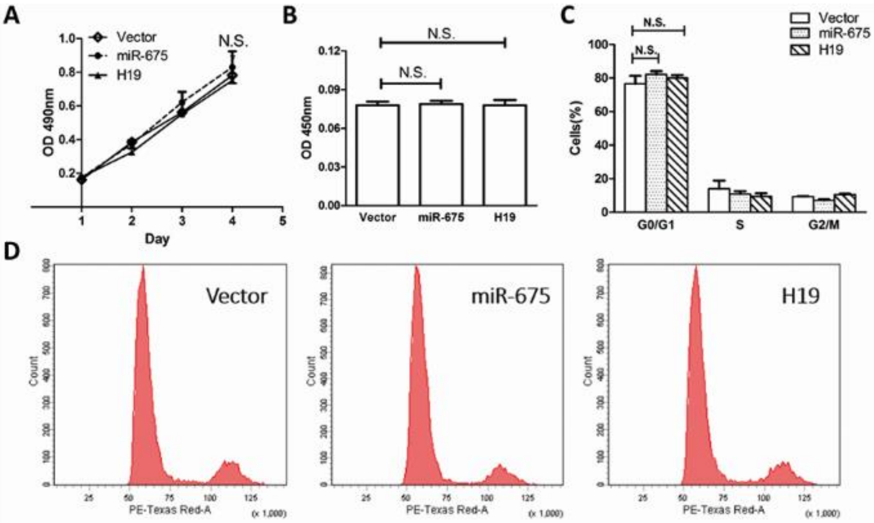

Supplementary Figure 5. Neither H19 nor miR-675 could affect cell cycle progression. (A&B) MTT assay (A) and BrdU incorporation assay (B) displayed that overexpression of H19 or miR-675 did not affect cell proliferation. (C) Cell flow cytometry assay exhibited that neither H19 nor miR-675 influenced cell cycle transition.

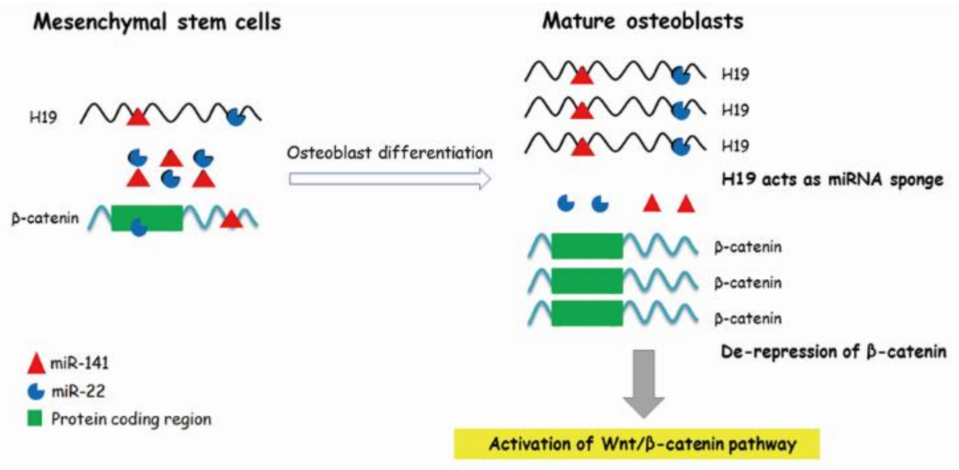

Supplementary Figure 6. Schematic overview of H19 regulatory network in osteoblast differentiation. LncRNA H19 shares two miRNA-response elements with β-catenin. In osteoblast cells, upregulated H19 serves as a natural miRNA sponge for miR-141 and miR-22, both of which target β-catenin. And increased H19 expression counteracts miRNA-mediated inhibition on β-catenin, resulting in activation of Wnt/β-catenin pathway and enhanced osteoblast differentiation.
